# Cultivar-specific physiological and proteomic patterns underlying mango susceptibility to bacterial black spot disease

**DOI:** 10.3389/fpls.2026.1911657

**Published:** 2026-08-25

**Authors:** Xiaona Xie, Dongting Li, Xianbin Hou, Qiufei Ouyang, Zhengjie Zhu, Xi Li, Zhengzhou Yang, Muzammil Hussain

**Affiliations:** 1 Guangxi Key Laboratory of Biology for Mango, College of Agriculture and Food Engineering, Baise University, Baise, China; 2 University Engineering Research Center for Preservation and Comprehensive Utilization of Subtropical Characteristic Agricultural Products in Guangxi, College of Agriculture and Food Engineering, Baise University, Baise, China; 3 Chongqing Three Gorges University, Chongqing, China

**Keywords:** bacterial black spot, comparative proteomics, disease resistance, mango, oxidative stress, photosynthesis

## Abstract

Mango bacterial black spot (MBBS) disease caused by *Xanthomonas citri* pv. *mangiferaeindicae* (Xcm) is a global problem that threatens mango productivity and quality worldwide. However, our understanding of mango physiological and proteomic responses to Xcm infection on leaves in different cultivars remains limited. Herein, we explored how two mango cultivars (Guifei and Aomang) respond to Xcm challenge at the physiological, biochemical, and proteomic levels at 6 and 12 ad post-inoculation. Our results revealed that Xcm infection induced progressive disease development in both cultivars as Guifei showed larger lesions and higher disease severity than Aomang. We further observed stronger induction of defense-related enzymes peroxidase, catalase and phenylalanine ammonia-lyase activities in Aomang, suggesting enhanced antioxidant and defense capacity against Xcm colonization. In addition, we observed decreased chlorophyll, soluble sugar, and reducing sugar contents, but increased hydrogen peroxide, superoxide, and malondialdehyde levels, pointing to oxidative stress and impaired photosynthetic activity. Proteomics analysis further showed cultivar-specific alteration in proteins abundance associated with primary metabolism, plant-pathogen interaction, hormone signaling, MAPK signaling, phenylpropanoid biosynthesis, and glutathione metabolism. Notably, Aomang showed early accumulation of defense-associated proteins, including receptor-like kinases, disease-resistance related proteins, hormone signaling components, and phenylpropanoid-associated enzymes, indicating early activation of immune-related pathways before subsequent metabolic suppression during prolonged infection. Overall, Guifei displayed greater disease susceptibility accompanied by early suppression of metabolic pathways, whereas Aomang exhibited reduced disease severity associated with enhanced antioxidant enzyme activation and early induction of defense-related proteins before broad pathway downregulation at late infection stage. Our findings integrating physiological and proteomic data revealed cultivar-specific changes in key metabolic pathways and provide a basis for targeted breeding or biotechnological approaches to manage MBBS disease.

## 1 Introduction

Mango (*Mangifera indica* L.) is an evergreen tree with fruits rich in nutritional value, containing large amounts of vitamins, minerals, and other substances beneficial to human health (Maldonado-Celis et al., 2019). Due to its multiple nutrients and high economic value, mango is found in tropical and subtropical regions of the world and regarded as the “King of Tropical Fruits” (Zhou et al., 2025). In China, mangoes have a wide cultivation area, with concentrated production areas in six provinces: Hainan, Guangxi, Guangdong, Yunnan, Fujian, and Sichuan (Guo et al., 2021). In recent years, with increasing consumer demand for processed mango products, the planting area has been expanding (Testa et al., 2018). However, at the same time, the fruit drop rate due to infection accounts for a high proportion of diseased fruits, seriously reducing fruit quality, market value and overall yield (Hussain et al., 2026). However, mango cultivars exhibit considerable variation in their responses to pathogen invasion, suggesting that cultivar-specific physiological and molecular mechanisms may determine disease progression and resistance outcomes. Understanding these differences is essential for identifying resistance-associated traits and developing effective strategies for disease management.

Among the major mango diseases, bacterial black spot, also known as mango angular leaf spot or bacterial canker, is considered a destructive disease (Sossah et al., 2024). Mango bacterial black spot (MBBS) pathogen *Xanthomonas citri* pv. *mangiferaeindicae* (Xcm) is particularly prevalent in mango-growing areas of China, including Guangxi (Bie et al., 2022). MBBS infects multiple parts of the mango plant, mainly the leaves, stems, and fruits. Mango leaves are the primary infection source for mango bacterial angular spots (Sossah et al., 2024). The main characteristic symptoms on leaves include small, water-soaked, dark green spots that gradually expand and even penetrate leaf veins. The leaf veins become obstructed, causing the multi-angled shape of the lesions to gradually split, forming dark brown lesion rings with a yellow halo around them. When MBBS infects mango fruits, initially irregular, dark green, sunken, small lesions of varying sizes form on the fruit surface. As the disease progresses, these lesions increase in size and eventually develop into crater-like dark brown cankerous spots. The lesions crack longitudinally, oozing a sticky bacterial exudate, resulting in an ugly fruit appearance and poor taste, severely affecting the economic value of mango (Sossah et al., 2024). Importantly, disease development following Xcm infection is influenced not only by pathogen virulence but also by the capacity of host plants to activate early defense responses (Timilsina et al., 2020). Therefore, comparative evaluation of symptom progression and host defense activation among mango cultivars provide valuable insights into mechanisms underlying resistance or susceptibility.

During pathogen invasion of the host, plant tissues undergo rapid physiological, biochemical, and microbiome shifts (Hussain et al., 2024; Hussain et al., 2025). For example, pathogen infection initially induces an oxidative burst in the plant, leading to excessive buildup of reactive oxygen species (ROS), including hydrogen peroxide (Sahu et al., 2022). During pathogen invasion, plants rapidly activate antioxidant defense systems to regulate ROS accumulation and maintain cellular redox balance. Enzymes such as catalase (CAT), peroxidase (POD), and superoxide dismutase (SOD) function not only in ROS detoxification but also contribute to pathogen-induced signaling processes associated with defense activation (Ramzan et al., 2021; Rajput et al., 2021). In addition, phenylalanine ammonia-lyase (PAL), a key enzyme in phenylpropanoid metabolism participates in the biosynthesis of antimicrobial compounds and structural barriers that restrict pathogen colonization (Vogt, 2010). Meanwhile, bacterial infection often triggers chlorophyll breakdown and inhibits photosynthetic capacity, signaling chloroplast dysfunction, and impaired physiological plant health. Together, alterations in hydrogen peroxide levels, antioxidant defense systems, and chlorophyll content provide useful insights into disease severity and the effectiveness of host defense responses (Cheaib and Killiny, 2025). Xcm, a hemibiotrophic bacterial pathogen, affects cellular metabolism and inhibits the defense response of mango by utilizing the secretion system and virulence effectors, leading to successful colonization and disease development (Liu et al., 2023).

Omics technologies have advanced considerably, offering new insights into how plants respond to pathogen infections at the molecular level (Kumar et al., 2025). In particular, proteomics approaches enable the identification of defense-associated proteins involved in pathogen recognition, receptor-mediated signaling, hormone regulation, secondary metabolism and redox regulation (Liu et al., 2023). Such information is critical for understanding why some cultivars rapidly activate defense mechanisms while others exhibit progressive metabolic disruption following bacterial infection. For example, our previous study on mango response to *Alternaria alternata* infection found a major shift in proteins linked to photosynthesis, phenylpropanoid, flavonoid biosynthesis, as well as the phenylalanine metabolism pathway (Xie et al., 2025). In another recent study involving both susceptible and resistant mango cultivars, early infection by the MBBS pathogen induced changes in the abundance of proteins associated with hormone signaling, redox homeostasis, and glutathione metabolism (Liu et al., 2023). Despite recent progress, very little is known about how physiological and proteomic profiles shift during the extended infection period in different mango cultivars following *Xanthomonas* challenge. To address this, the present study compared healthy and *Xanthomonas* infected leaf samples from two different mango cultivars at 6 and 12 ad post-infection for physiological parameters alongside untargeted proteomic profiling. We hypothesized that both mango cultivars would undergo significant physiological and proteomic alterations upon *Xanthomonas* challenge. Specifically, we proposed that (a) *Xanthomonas* infection would intensify oxidative stress resulting in increased hydrogen peroxide accumulation, (b) bacterial infection would impair photosynthetic performance by lowering chlorophyll content, and (c) proteomic profiling would reveal cultivar-specific regulation of proteins involved in stress response and defense signaling, thereby providing molecular insights into differential resistance mechanisms against Xcm. The integration of physiological and proteomic analyses in the current study would help highlight key aspects of the possible mango resistance pathways to bacterial infection.

## 2 Materials and methods

### 2.1 Plant material and bacterial pathogen

Healthy mango seedlings belonging to two cultivars, ‘Guifei’ and ‘Aomang’ were purchased from a nursery located in Sitang Town, Youjiang District, Baise City, Guangxi, China. Mango seedlings were cultivated in barrels with a growth substrate composed of a mixture of soil, cultivation substrate, and organic fertilizer in a ratio 5:3:2 (v/v/v). Water and fertilizer were applied according to standard nursery practices. The bacterial pathogen used in this study was *X. citri* pv. *mangiferaeindicae* (Xcm) strain B3. This strain was previously isolated from diseased mango leaves exhibiting typical bacterial black spot symptoms collected from commercial orchards in Baise, Guangxi, China. The complete genome sequence, taxonomic identification, isolation procedure, and basic biological characteristics of strain B3 have been fully characterized and published in our earlier study (Ouyang et al., 2025). For the experiment, the preserved strain B3 was recovered and cultured under consistent conditions as described in the previous genome report to ensure experimental consistency and reproducibility.

When the mango seedlings reached the 8–10 leaf stage, an Xcm bacterial suspension was applied to the abaxial surface of the leaves using sterile cotton swabs. After 6 and 12 ad post-inoculation, lesion diameters were recorded, and disease severity was assessed. The 6 and 12 ad sampling points were selected to compare two sequential stages of established disease progression. The 6 ad timepoint represented the earlier stage, when visible lesions and measurable host responses were present, whereas 12 ad represented a more advanced stage associated with greater lesion development and prolonged pathogen challenge. Each leaf was individually assigned a disease severity grade based on its lesion diameter using a grading system adapted from Xiaomei et al. (2009). For subsequent physiological assays, leaves samples from three independent mango seedlings were collected at 0, 6, and 12 ad post-inoculation and immediately frozen at -80 °C. For ultra-fast quantitative proteomics analysis, mango leaves were flash-frozen in liquid nitrogen and sent to Metware Biotechnology Co., Ltd.

### 2.2 Mango physiological responses to bacterial inoculation

Mango leaf chlorophyll content was measured using an MPM-100 *in-situ* plant multi-pigment meter (Shi et al., 2025). The clip of the MPM-100 m was used to clamp the healthy and infected areas of the leaf for reading, and the data were recorded. The soluble sugar content was determined using the anthrone colorimetric method (Denova-Lozano et al., 2026). The absorbance value was measured, and the soluble sugar content in the leaves was calculated using a standard curve. The reducing sugar content was measured using the 3,5-dinitrosalicylic acid DNS colorimetric method (Saqib and Whitney, 2011). Briefly, 0.5 ag of mango leaf sample was homogenized in a small amount of distilled water, and then adjusted to 50 mL. The solution was incubated in a constant-temperature water bath at 50 °C for 10 min to extract reducing sugars and then centrifuged at 4000 rpm for 10 min to obtain the supernatant. Next, 2 mL of the reducing sugar extract was mixed with DNS reagent, heated in a boiling water bath for 5 min, cooled to room temperature, diluted to 25 mL, and the absorbance was measured at 540 nm. A glucose standard curve was used to measure the concentration of reducing sugar in the leaf samples. Hydrogen peroxide content was measured in mango leaves according to the method described by Patterson et al. (1984). Briefly, the leaf tissue was homogenized in the presence of 5% TCA (5 ml) and 0.15 ag of activated charcoal and the mixture was centrifuged at 10000 rpm for 20 min. Next, the supernatant was mixed with 8 µg catalase and incubated at room temperature for 10 min. Afterwards, 1 ml of colorimetric reagent (10 mg of 4-aminoantipyrine, 10 mg of phenol, 5 mg of peroxidase (150 U mg^–1^), dissolved in 50 ml of 100 mM acetic acid buffer) was added and the solution was then incubated for 10 min at 30 °C. The hydrogen peroxide content was measured using spectrophotometry at an absorbance of 505 nm. The superoxide radical (O_2_^−^·) content was measured using the tetramethylbenzidine (TMB) chromogenic method. The malondialdehyde (MDA) content was determined following the thiobarbituric acid (TBA) method. Leaf sample were homogenized with trichloroacetic acid (TCA) and quartz sand and centrifuged. Next, 2mL of the supernatant was mixed with TBA reagent, heated in a boiling water bath for 15 min, rapidly cooled, and centrifuged. The absorbance of the reaction was measured at 450, 532, and 600 nm. The activities of superoxide dismutase (SOD), peroxidase (POD), catalase (CAT), and phenylalanine ammonia-lyase (PAL) in leaves were measured using commercial assay kits (Suzhou Keming Biotechnology Co., Ltd., China). Each measurement was performed using three independent biological replicates per treatment.

### 2.3 Protein extraction, liquid chromatography-mass spectrometry, and data processing

Mango leaf samples from three independent mango seedlings were collected and ground to a fine powder in liquid nitrogen and then transferred to a 1.5 mL tube and mixed with L3 lysis buffer. The mixture was sonicated on ice for 10 min and centrifuged to collect the supernatant (protein extract). Cold acetone was added to the supernatant and proteins were precipitated overnight at –20°C. After centrifugation at 4°C, the resulting pellet was washed three times with cold acetone and then re-dissolved in 8 M urea. The total protein concentration was determined using a BCA kit (Beyotime, Shanghai, China). For each sample, 100 μg of protein was taken, and the volume was adjusted to 200 μL with 8 M urea. Proteins were reduced with 5 mM DTT at 37°C for 45 min and alkylated with 11 mM iodoacetamide (IAM) in the dark at room temperature for 15 min. Subsequently, 800 μL of 25 mM ammonium bicarbonate and 3 μL of trypsin (Promega) were added, and digestion was performed overnight at 37°C. The resulting peptides were acidified with 20% TFA to pH 2–3 and desalted using a C18 column (Millipore, Billerica, MA, USA). The peptide concentration was determined using the Pierce™ Quantitative Peptide Detection Kit (Thermo Fisher Scientific).

The peptide samples were separated using a NanoElute UHPLC system at a flow rate of 500 nL/min. Mobile phase A was 0.1% formic acid in water, and mobile phase B was 0.1% formic acid in 100% acetonitrile. A total of 200 ng of peptides were loaded onto an analytical column (IonOpticks, Australia, 15 cm × 75 μm, 1.6 μm C18) maintained at 50°C. The LC gradient was as follows: 0–17 min, 5–25% B; 17–18 min, 25–40% B; 18–19 min, 40–95% B; and 19–20 min, held at 95% B. The separated peptides were analyzed using a timsTOF Pro2 mass spectrometer (Bruker) in the positive ion mode. The precursor m/z range was 100–1700, and ion mobility (1/K_0_) ranged from 0.85 to 1.3 Vs/cm². The accumulation and release times were 100 ms with nearly 100% ion utilization. The capillary voltage was set to 1500 aV, drying gas flow to 3 L/min, and drying temperature to 180°C. For ddaPASEF acquisition, four MS/MS scans were performed per cycle (total cycle time 0.53 s), with charge range 0–5, dynamic exclusion time 0.4 min, target intensity 10,000, and intensity threshold 1500. Collision energy increased linearly with ion mobility (27 eV at 1/K_0_ = 0.85 Vs/cm² and 45 eV at 1/K_0_ = 1.3 Vs/cm²). The quadrupole isolation width was 2 Th for m/z < 700 and 3 Th for m/z > 800. For diaPASEF, the mass range was 400–1200, mobility range 0.85–1.3 Vs/cm², mass width 12 Th, mass overlap 0.1, 24 mass steps per cycle, and 2 mobility windows, giving a total of 48 acquisition windows. The average cycle time was 1.17 s.

Raw DIA data were processed using DIA-NN (version 1.8.1) in library-free mode. The protein database used was NCBI_txid29780_mangguo_20240519.fasta (53,829 sequences). Deep learning-based spectral library prediction was enabled, and match-between-runs (MBR) were used to generate a spectral library from the DIA data for re-analysis. Both precursor and protein false discovery rates (FDR) were set to 1%. Protein quantification was performed using the MaxLFQ algorithm implemented in DIA-NN. For each protein, the median log_2_ ratio of peptide intensities between samples was used to estimate the relative protein abundance. After quantification, within-sample normalization was applied: the relative abundance R_ij_ for protein j in sample i was calculated as R_ij_ = I_ij_/median(I_i_), where I_ij_ is the raw intensity. Differential expression analysis was performed between groups with biological replicates. The fold change (FC) was calculated as the mean intensity ratio between the groups. Statistical significance was assessed using Student’s t-test (for two groups) or ANOVA (for more than two groups). P-values were adjusted using the Benjamini–Hochberg method for multiple testing, where appropriate.

### 2.4 Bioinformatics analysis

Identified and differentially expressed proteins were functionally annotated using Gene Ontology (GO), KOG functional classification, KEGG pathway analysis, protein domain analysis, subcellular localization prediction, and signal peptide prediction. Enrichment analysis of GO terms, KOG categories, KEGG pathways, and protein domains was performed using a hypergeometric test to identify significantly enriched terms among differentially expressed proteins relative to the background (all identified proteins).

## 3 Results and analysis

### 3.1 Disease progression and defense enzyme responses to bacterial black spot pathogen

**Figure 1:**
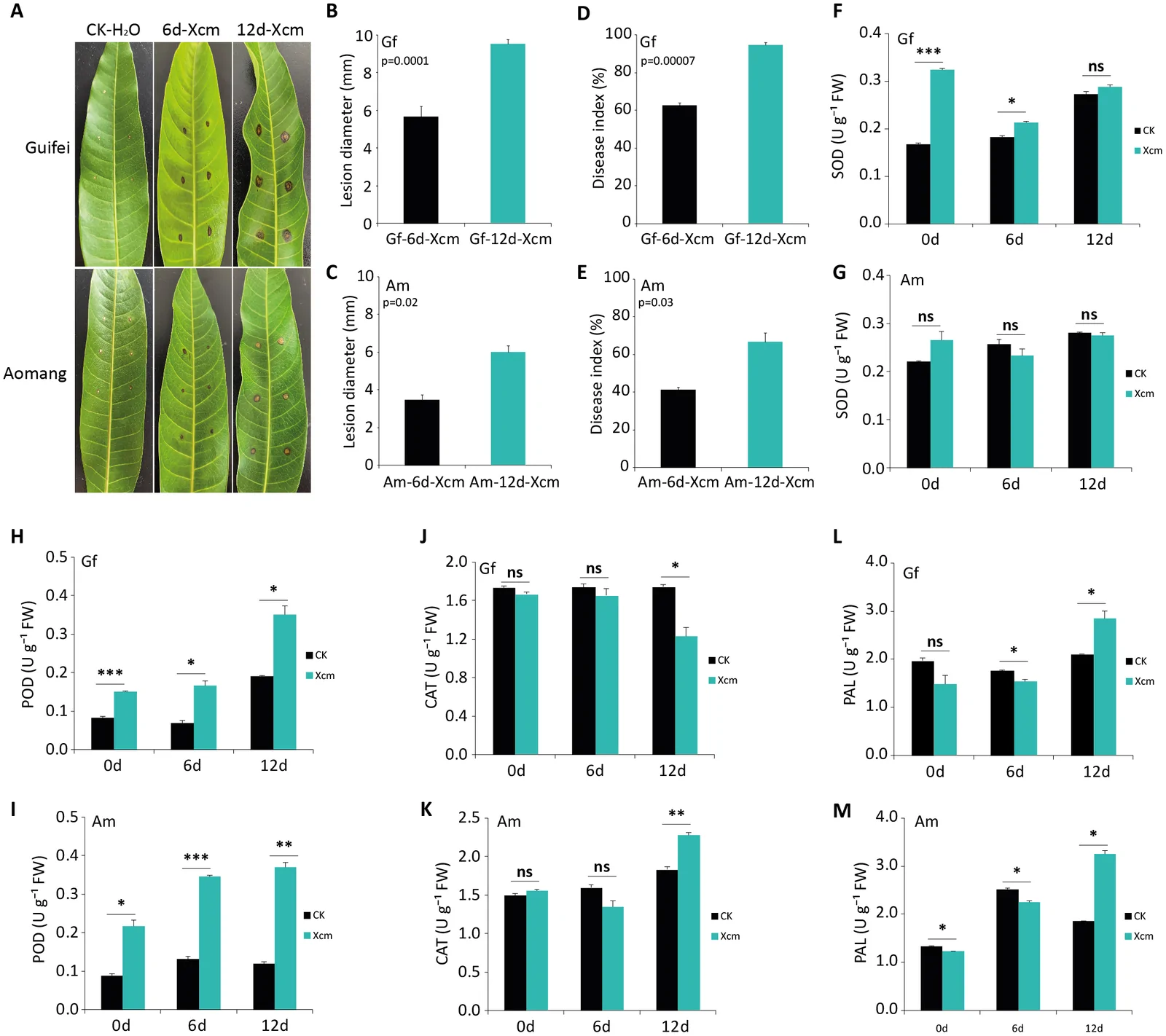
Disease progression and antioxidant and defense-related enzyme responses in two mango cultivars following Xcm inoculation. **(A)** Representative images of leaves from Guifei (Gf) and Aomang (Am) treated with sterile water (CK-H_2_O) or inoculated with Xcm for 6 ad and 12 ad. Lesion diameter of Xcm infection in Gf leaves **(B)** and Am leaves **(C)**. Disease index of Xcm infection in Gf **(D)** and Am **(E)** cultivars. **(F, G)** Superoxide dismutase (SOD) activity; **(H, I)** peroxidase (POD) activity; **(J, K)** catalase (CAT) activity; and **(L, M)** phenylalanine ammonia-lyase (PAL) activity in Gf and Am leaves at 0, 6, and 12 ad. Black and turquoise bars represent the control (CK) and Xcm-inoculated treatments, respectively. Data are presented as the mean ± SE (n=3). The p value in panel B-E indicate differences between 6 and 12 ad within each cultivar. In panel F-M, statistical significance between CK and Xcm treatment at each sampling point was determined using Student’s t-test. ns, no significant difference; *P < 0.05, **P < 0.001, ***P < 0.0001.

Representative leaf images showed visible disease symptoms developed progressively in both mango cultivars following inoculation with Xcm, whereas sterile water treated leaves remained symptomless (**Figure 1A**). Guifei (Gf) leaves exhibited larger and more prominent necrotic lesions than Aomang (Am) leaves at 6 ad post-inoculation, and symptoms severity increased substantially in both cultivars by 12 ad post-inoculation. In Gf, lesion diameter increased from 5.66 mm at 6 ad to 9.53 mm at 12 ad post-inoculation (p = 0.0001; **Figure 1B**), while the disease index increased from 62% to 94% (p = 0.00007; **Figure 1D**). In Am, lesion diameter increased from 3.46 mm to 6 mm (p = 0.02; **Figure 1C**), accompanied by an increase in the disease index from 41% to 66% (p = 0.03; **Figure 1E**). The consistently greater lesion diameter and disease index observed in Gf indicated a higher susceptibility to Xcm than that of Am.

Xcm inoculation differentially affected antioxidant and defense-related enzyme activities in the two mango cultivars. In Gf, SOD activity was significantly higher in Xcm-inoculated leaves than CK at 0 ad and 6 ad, whereas no significant difference was detected at 12 ad (**Figure 1F**). In contrast, SOD activity in Am did not differ significantly between CK and inoculated leaves at any sampling time (**Figure 1G**). POD activity was significantly induced by Xcm in both cultivars. In Gf, POD activity was higher in inoculated leaves at 0, 6 and 12 ad, with the highest activity recorded at 12 ad (**Figure 1H**). Am exhibited a similar but more pronounced response with significantly elevated POD activity at all sampling points, particularly at 6 and 12 ad post-inoculation (**Figure 1I**).

CAT activity displayed contrasting late-stage responses in the two cultivars. In Gf, CAT activity was not significantly affected by Xcm at 0 and 6 ad but was significantly reduced at 12 ad (**Figure 1J**). In Am, CAT activity remained unchanged at 0 and 6 ad but increased significantly in inoculated leaves at 12 ad (**Figure 1K**). PAL activity also exhibited a time-dependent response. In Gf, PAL activity was unaffected at 0 ad, significantly reduced at 6 ad, and markedly increased at 12 ad following Xcm inoculation (**Figure 1L**). In Am, PAL activity was significantly lower in inoculated leaves at 0 and 6 ad but increased substantially above the control level at 12 ad (**Figure 1M**). Overall, the stronger late-stage induction of POD, CAT and PAL activities in Am coincided with its lower lesion development and disease index relative to Gf.

### 3.2 Changes in chlorophyll, soluble sugar and reducing sugar contents of two mango cultivars infected with bacterial black spot pathogen

**Figure 2:**
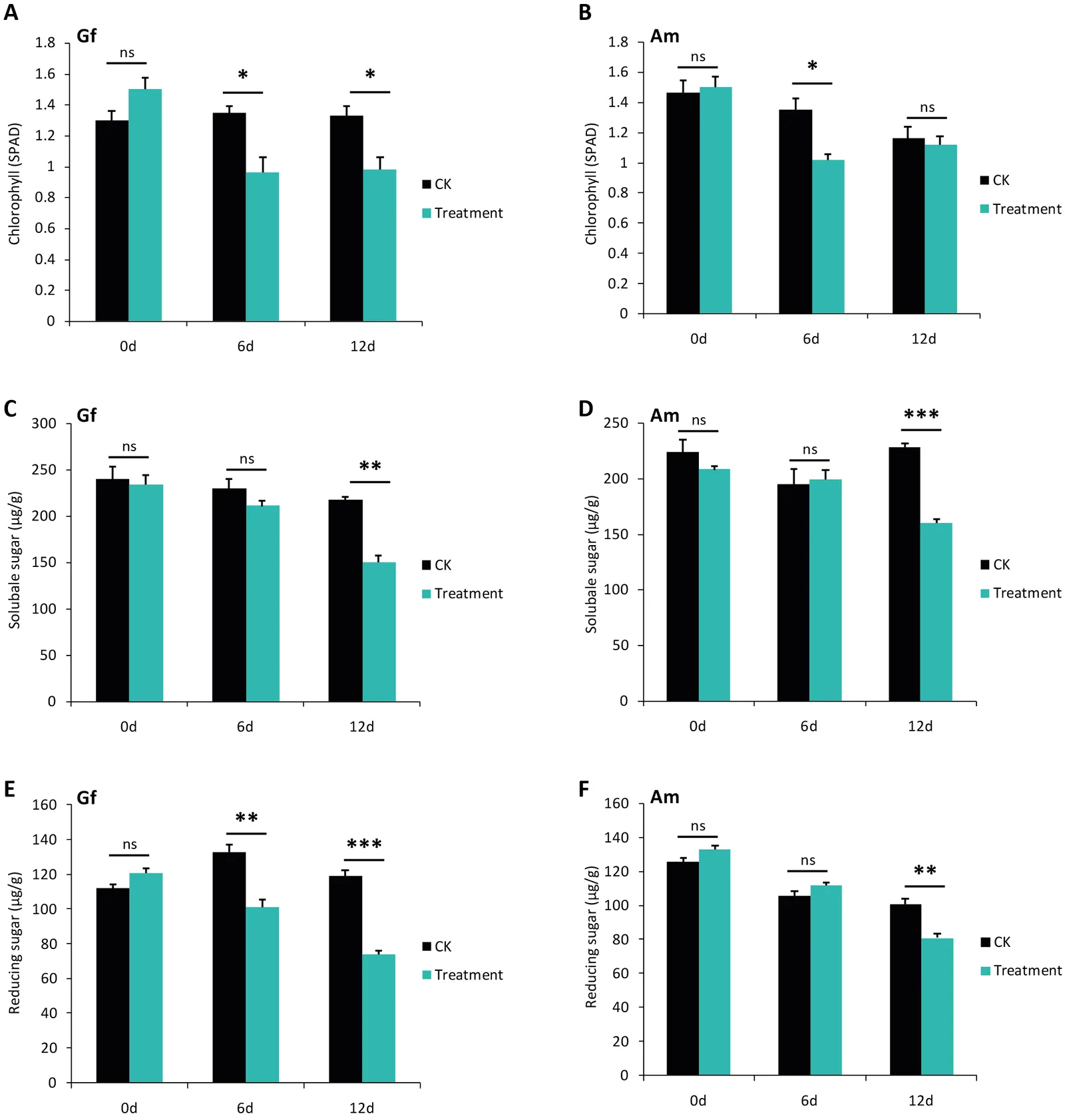
Dynamic alterations in chlorophyll, soluble sugar, and reducing sugar contents in leaves of two mango cultivars (Guifei, Gf; Aomang, Am) at 0 ad, 6 ad, and 12 ad after inoculation with bacterial black spot pathogen. **(A, B)** Chlorophyll content; **(C, D)** Soluble sugar content; **(E, F)** Reducing sugar content. Black bars represent untreated control (CK), blue bars represent the pathogen-inoculated treatment group. Data are presented as the mean ± SE (n=3). Statistical significance between CK and Xcm treatment at each sampling point was determined using Student’s t-test. ns, no significant difference; *P < 0.05, **P < 0.001, ***P < 0.0001.

Following the assessment of disease development and defense-enzyme activities, we examined changes in photosynthetic pigments and carbohydrate-related traits. At the initial sampling point of 0 ad, no statistically significant differences in chlorophyll, soluble sugar, and reducing sugar contents were observed between the control and pathogen-inoculated treatments for mango cultivars Guifei and Aomang (**Figures 2A–F**). However, a significant decrease in chlorophyll content was observed at both 6 ad and 12 ad post-inoculation in Guifei, whereas Aomang only displayed a prominent decrease in chlorophyll in pathogen-treated leaves at 6 ad (**Figures 2A, B**). For soluble sugar content, both cultivars showed non-significant differences at 6 ad, but pathogen inoculation caused a significant decrease in soluble sugar at 12 ad (**Figures 2C, D**). With respect to reducing sugars, we observed a significant decrease only in Guifei, but not in Aomang leaves at 6 ad, but the reducing sugar levels were markedly reduced in pathogen-challenged leaves at 12 ad for both mango cultivars (**Figures 2E, F**). Overall, infection by the bacterial black spot pathogen induced gradual depletion of leaf chlorophyll and sugar, but the timing and magnitude of significant physiological suppression varied between Guifei and Aomang cultivars.

### 3.3 Accumulation of hydrogen peroxide, superoxide radical, and malondialdehyde in pathogen-inoculated leaves of two mango cultivars

**Figure 3:**
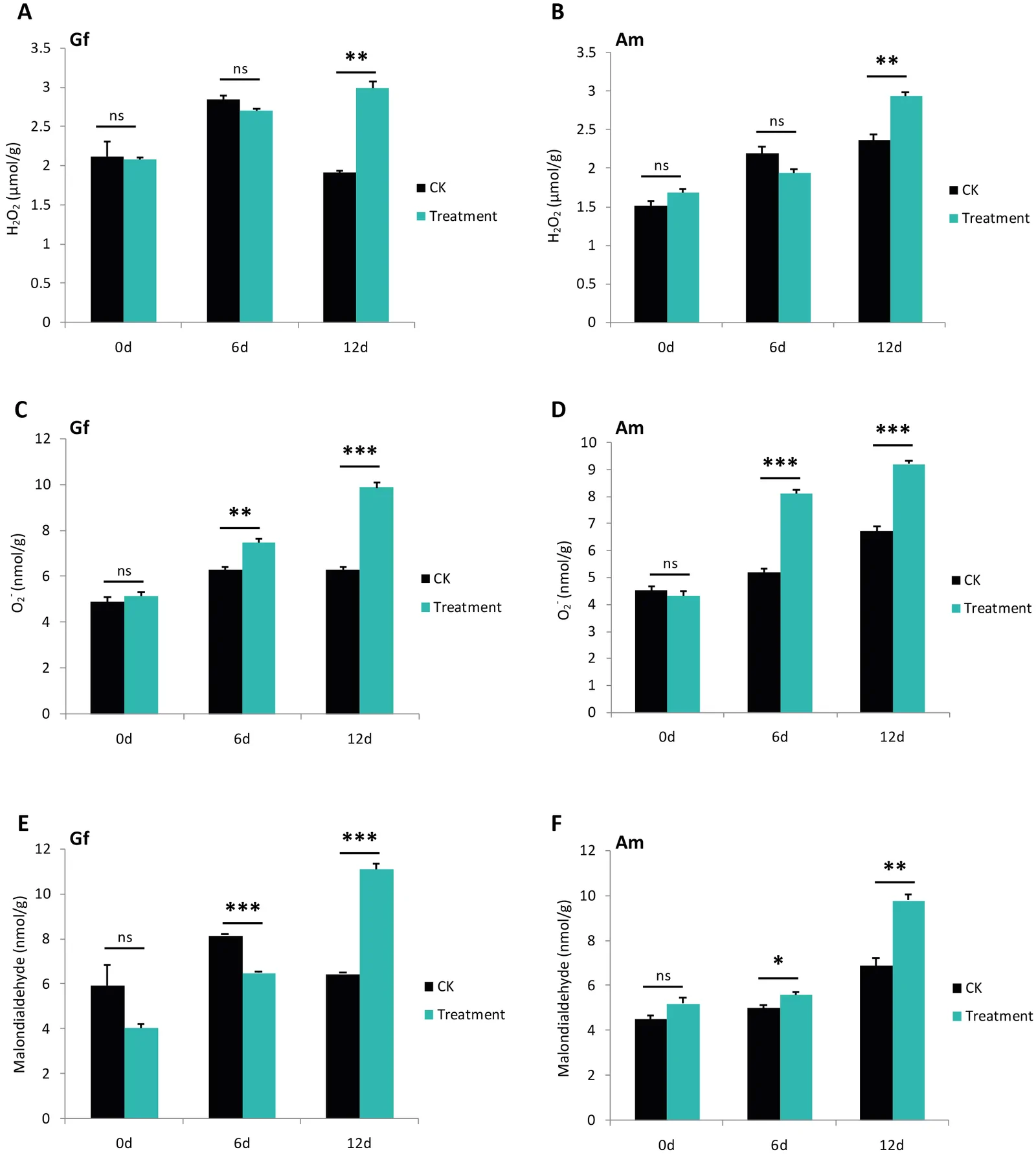
Dynamic changes in H_2_O_2_, O_2_^-^, and malondialdehyd (MDA) concentrations in leaves of two mango cultivars (Guifei, Gf; Aomang, Am) at 0, 6, and 12 ad after inoculation with bacterial black spot pathogen. **(A, B)** H_2_O_2_ content; **(C, D)** O_2_^-^ content; **(E, F)** MDA content. Black bars represent untreated control (CK), blue bars represent the pathogen-inoculated treatment group. Data are presented as the mean ± SE (n=3). Statistical significance between CK and Xcm treatment at each sampling point was determined using Student’s t-test. ns, no significant difference; *P < 0.05, **P < 0.001, ***P < 0.0001.

At the initial sampling point of 0 ad, no statistically significant differences in H_2_O_2_, O_2_^-^, and MDA concentrations were detected between the treatment and CK for both mango cultivars (**Figures 3A–F**). For H_2_O_2_ content, Guifei and Aomang showed non-significant differences at 6 ad, but a significant increase was observed in the pathogen-inoculated leaves at 12 ad (**Figures 3A, B**). In terms of O_2_^-^ content, pathogen inoculation induced significantly higher O_2_^-^ accumulation at 6 and 12 ad in both mango cultivars (**Figures 3C, D**). Regarding MDA, Guifei mango showed a significant decrease in MDA levels in pathogen-challenged leaves at 6 ad but a dramatic increase at 12 ad (**Figure 3E**). While, MDA levels increased significantly at both 6 and 12 ad in Aomang (**Figure 3F**). Overall, pathogen infection boosted the production of H_2_O_2_, O_2_^-^, and MDA in mango leaves.

### 3.4 Identification of mango differentially abundant proteins in response to *X. citri pv. mangiferaeindicae*

**Figure 4:**
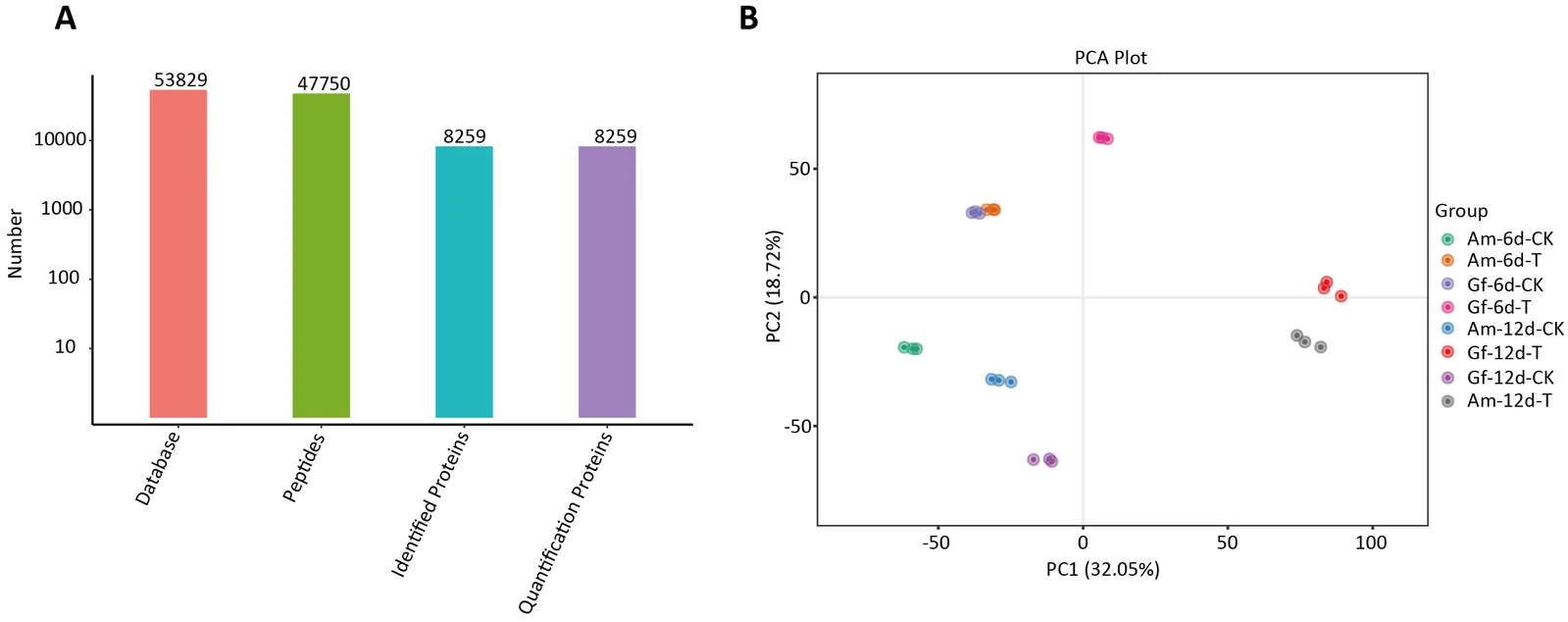
Overview of the proteome dataset. **(A)** Summary of MS/MS spectrum search analysis revealed numbers of total spectrums in database, peptides, identified proteins, and quantifiable proteins. **(B)** PCA plot showing the variation in the differential proteins between pathogen-infected (T) and control (CK) samples at different time points (6 and 12d) from two mango cultivars (Gf, Guifei; Am, Aomang).

Proteomic profiling revealed 47750 unique matched peptides, which enabled the identification of 8259 non-redundant proteins across the full sample set (**Figure 4A**). Principal component analysis demonstrated that PC1 and PC2 explained 32.05% and 18.72% of total proteomic variation, respectively, together accounting for over half of the total dataset variance (**Figure 4B**). All replicates belonging to the same experimental group were clustered compactly in a PCA coordinate plot. Meanwhile, a clear separation was observed for samples grouped by infection duration (6 and 12 ad) and treatment status (CK and T), confirming that bacterial pathogen infection triggered widespread remodeling of the global proteome.

**Figure 5:**
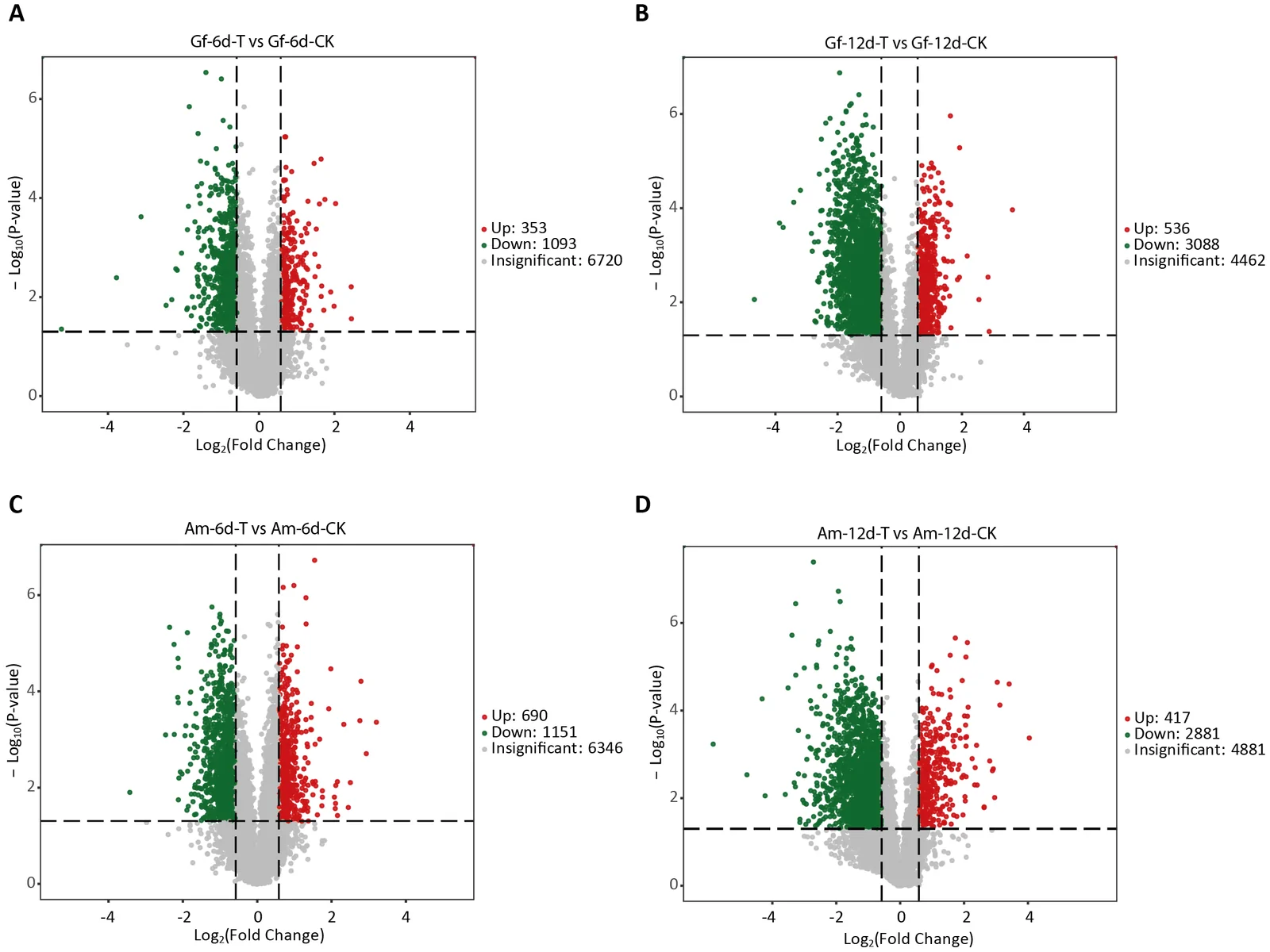
Volcano plots illustrating differentially abundant proteins (DAPs) from proteome analysis of two mango cultivars (Guifei, Gf; Aomang, Am) at 6 ad and 12 ad of pathogen inoculation (T) compared to the untreated control (CK). **(A)** Gf-6d-T versus Gf-6d-CK; **(B)** Gf-12d-T versus Gf-12d-CK; **(C)** Am-6d-T versus Am-6d-CK; and **(D)** Am-12d-T versus Am-12d-CK. Red dots represent significantly upregulated DAPs, green dots represent significantly downregulated DAPs, and grey dots indicate proteins with non-significant abundance changes.

To characterize bacterial pathogen-induced proteomic changes in mango leaves, differentially abundant proteins (DAPs) were identified between the pathogen-inoculated and control groups across the two mango cultivars at 6 ad and 12 ad post-treatment (**Figures 5A–D**). In the Guifei at 6 ad (Gf-6d-T vs. Gf-6d-CK), 353 proteins were significantly increased in abundance, while 1093 proteins were significantly decreased upon bacterial pathogen infection (**Figure 5A**; **Supplementary Figure 1**). By 12 ad post-inoculation (Gf-12d-T vs. Gf-12d-CK), the total number of DAPs markedly expanded to 596 upregulated and 3088 downregulated proteins (**Figure 5B**; **Supplementary Figure 2**). For Aomang at the 6 ad time point (Am-6d-T vs. Am-6d-CK), 690 proteins were significantly upregulated and 1151 proteins were significantly downregulated upon bacterial pathogen infection (**Figure 5C**; **Supplementary Figure 3**). At 12 ad (Am-12d-T vs. Am-12d-CK), the count of upregulated proteins declined to 417, while downregulated proteins rose sharply to 2881 (**Figure 5D**; **Supplementary Figure 4**). Across all four pairwise comparisons, proteins with significantly reduced abundance consistently outperformed those with induced accumulation, indicating that pathogen infection elicited widespread global suppression of the mango proteome.

### 3.5 Functional classification of mango DAPs based on gene ontology annotation

Gene Ontology (GO) functional classification of differentially abundant proteins (DAPs) from two mango cultivars (Guifei and Aomang) in response to bacterial pathogen infection across two sampling timepoints revealed DAPs annotated into biological process (BP), cellular component (CC), and molecular function (MF). For the BP category, cellular and metabolic processes consistently constituted the two most abundant annotated functional groups for both Guifei and Aomang at 6 and 12 ad (**Supplementary Figures 5**, **6**). In addition, categories including response to stimulus, biological regulation, and localization were moderately abundant at both sampling stages. Within the CC compartment, cellular anatomical entity and protein-containing complex represented the top two highly annotated terms, exhibiting striking increases in total downregulated DAPs at 12 ad relative to 6 ad for both Guifei and Aomang (**Supplementary Figures 5**, **6**). For MF annotations, catalytic activity and binding dominated the molecular function profile, again displaying markedly amplified total DAPs abundance at 12 ad versus 6 ad for each cultivar (**Supplementary Figures 5**, **6**). Collectively, these GO classification outcomes demonstrate that the bacterial pathogen infection drives progressive, time-dependent global suppression of core metabolic, structural and catalytic proteomic programs in leaves of both mango genotypes from 6 ad to 12 ad, accompanied by cultivar-specific fine-tuning of differential protein expression.

### 3.6 KEGG pathway enrichment analysis of DAPs in two mango cultivars

**Figure 6:**
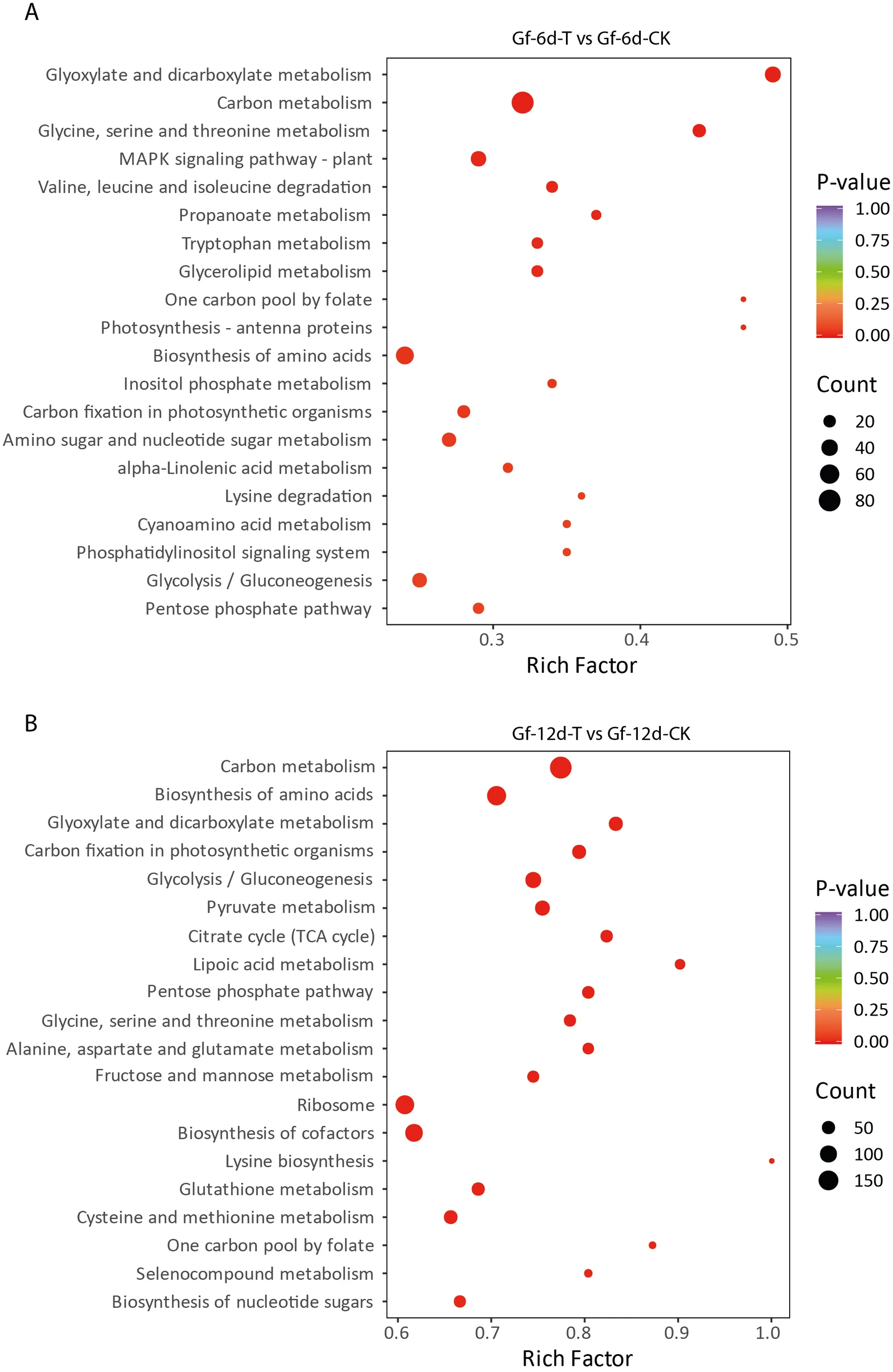
Top enriched KEGG pathways for DAPs from pathogen-challenged and control Guifei leaves at 6 ad **(A)** and 12 ad **(B)** post-inoculation. Size of the circle represents the number of proteins enriched in the pathways and color of the circle represents their p-values.

We first quantified the DAPs assigned to annotated KEGG functional pathways in the Guifei cultivar 6 and 12 ad after Xcm infection (**Supplementary Figure 7**). The principal pathways associated with primary metabolism included carbon metabolism, biosynthesis of amino acids, photosynthesis-antenna proteins, the MAPK signaling pathway, and glycolysis/gluconeogenesis. KEGG enrichment analysis revealed that the significantly enriched pathways at 6 ad included glyoxylate and dicarboxylate metabolism; carbon metabolism; glycine, serine and threonine metabolism; the MAPK signaling pathway; valine, leucine and isoleucine degradation; propanoate metabolism; tryptophan metabolism; glycerolipid metabolism; photosynthesis-antenna proteins; biosynthesis of amino acids; inositol phosphate metabolism; amino sugar and nucleotide sugar metabolism; alpha-linolenic acid metabolism; lysine degradation; phosphatidylinositol signaling system; cyanoamino acid metabolism; and glycolysis/gluconeogenesis (**Figure 6A**). At 12 ad, several pathways detected at 6 ad were no longer significantly enriched, whereas the citrate cycle (TCA cycle); lipoic acid metabolism; glutathione metabolism; cysteine and methionine metabolism; selenocompound metabolism; and biosynthesis of nucleotide sugar were newly identified among the significantly enriched pathways (**Figure 6B**). Examination of the direction of protein regulation showed that many DAPs assigned to these pathways were less abundant in infected leaves than in the control group.

At 6 ad of Xcm infection in the Guifei cultivar (Gf-6d-T vs. Gf-6d-CK), carbon metabolism showed a strong predominance of downregulated DAPs, comprising 77 downregulated proteins and 6 upregulated proteins (**Supplementary Figure 7A**; **Supplementary Excel File 1**). The upregulated proteins with high fold changes included glyoxylate/hydroxypyruvate reductase HPR3-like (XP_044493773.1), whereas the downregulated proteins included methylenetetrahydrofolate reductase 2-like (XP_044466196.1), glyceraldehyde-3-phosphate dehydrogenase (ADX97321.1), and A (XP_044504230.1; **Supplementary Excel File 1**). Similarly, the biosynthesis of amino acids pathway contained 45 downregulated DAPs including cytosolic enolase 3 (XP_044469600.1), pyruvate kinase, cytosolic isozyme-like (XP_044490158.1), and ornithine transcarbamylase (XP_044509902.1), compared with 5 upregulated DAPs including glutamate synthase 1 [NADH] (XP_044468679.1) and histidinol dehydrogenase (XP_044478194 glyceraldehyde-3-phosphate dehydrogenase.1; **Supplementary Excel File 1**). These results indicated a marked predominance of reduced protein abundance in central carbon and amino acid metabolic machinery in infected leaves relative to control leaves (**Supplementary Figure 7A**; **Supplementary Excel File 1**).

The MAPK signaling pathway contained 11 upregulated DAPs, including polygalacturonase inhibitor-like (XP_044480432.1) and probable WRKY transcription factor 50 (XP_044464688.1), and 23 downregulated DAPs, including probable transcription factor PosF21 (XP_044466455.1) and transcription factor MYC2-like (XP_044503291.1; **Supplementary Excel File 1**). The photosynthesis-antenna proteins pathway contained only downregulated DAPs, including chlorophyll a-b binding protein CP29.3 (XP_044462040.1), photosystem I chlorophyll a/b-binding protein 3-1, chloroplastic (XP_044489557.1), and chlorophyll a-b binding protein P4 (XP_044477011.1; **Supplementary Excel File 1**).

The inhibitory trend at 6 ad was markedly exacerbated at the 12 ad sampling point in Guifei (Gf-12d-T vs. Gf-12d-CK), with a substantial increase in total downregulated DAPs counts across the major functional pathways (**Supplementary Figure 7B**). Carbon metabolism contained 190 downregulated DAPs, including 3-hydroxyisobutyryl-CoA hydrolase-like protein 3 (XP_044464142.1), malate dehydrogenase (XP_044473925.1) and phosphoglycerate kinase (XP_044506154.1), compared with 9 upregulated DAPs, including ribulose bisphosphate carboxylase large chain, commonly known as Rubisco (XP_044466616.1) and ribose-5-phosphate isomerase 4 (XP_044479409.1; **Supplementary Excel File 1**). The biosynthesis of amino acid pathway comprised 139 downregulated DAPs, including bifunctional aspartokinase/homoserine dehydrogenase 1 (XP_044511708.1) and 5 upregulated DAPs, including cysteine synthase-like (XP_044484356.1) and 3-phosphoshikimate 1-carboxyvinyltransferase 2-like (XP_044503673.1; **Supplementary Excel File 1**).

The enriched TCA cycle pathway contained 1 upregulated DAP, dihydrolipoyllysine-residue acetyltransferase component 5 of pyruvate dehydrogenase complex (XP_044476166.1), and 44 downregulated DAPs, including malate dehydrogenase (XP_044473925.1), succinate--CoA ligase [ADP-forming] subunit beta (XP_044504205.1), and ATP-citrate synthase beta chain protein 2 (XP_044499912.1; **Supplementary Excel File 1**). Glutathione metabolism also contained 1 upregulated DAP, annotated as phospholipid hydroperoxide glutathione peroxidase (XP_044485629.1), and 51 downregulated DAPs, including glutathione S-transferase (XP_044498155.1), glutathione S-transferase U7-like (XP_044510475.1), and phospholipid hydroperoxide glutathione peroxidase 1 (XP_044471943.1; **Supplementary Excel File 1**), confirming progressive strengthening of infection-triggered metabolic repression over time in leaves (**Supplementary Figure 7B**).

**Figure 7:**
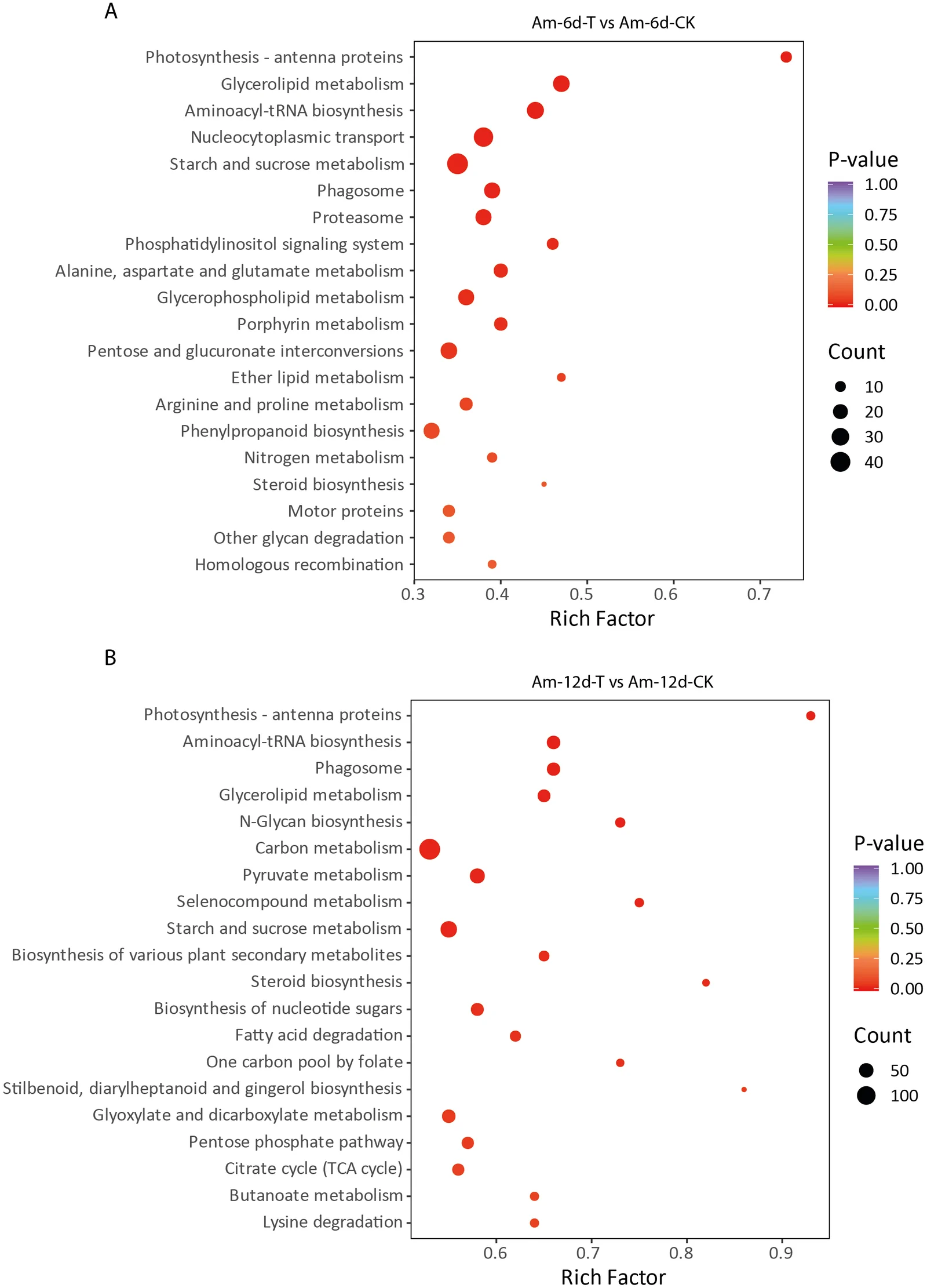
Top enriched KEGG pathways for DAPs from pathogen-challenged and control Aomang leaves at 6 ad **(A)** and 12 ad **(B)** post-inoculation. Size of the circle represents the number of proteins enriched in the pathways and color of the circle represents their p-values.

KEGG enrichment analysis of the Aomang cultivar further identified photosynthesis-antenna protein, glycerolipid metabolism, and aminoacyl-tRNA biosynthesis as enriched pathways at both sampling times (**Figures 7A, B**). However, a distinct pattern of DAP regulation under non-enriched pathways was observed in the Aomang cultivar at 6 ad (Am-6d-T vs. Am-6d-CK), after pathogen infection compared with Guifei (**Supplementary Figure 8A**). The plant–pathogen interaction pathway (13 downregulated vs. 41 upregulated DAPs), plant hormone signal transduction (18 downregulated vs. 19 upregulated DAPs), the MAPK signaling pathway (7 downregulated vs. 23 upregulated DAPs), pentose and glucuronate interconversions (7 downregulated vs. 19 upregulated DAPs), and phenylpropanoid biosynthesis (6 downregulated vs. 18 upregulated DAPs) showed a predominance of upregulated DAPs (**Supplementary Figure 8A**). In contrast, carbon metabolism showed a predominance of downregulated DAPs, similar to the pattern observed in Guifei, with 50 downregulated and 4 upregulated DAPs. The biosynthesis of amino acid pathway also contained predominantly downregulated DAPs with 41 down and 4 upregulated proteins in infected Aomang leaves (**Supplementary Figure 8A**).

Among the upregulated proteins with the highest fold changes assigned to the plant-pathogen interaction pathway were receptor-like protein kinase FERONIA (XP_044468609.1), disease resistance protein RPV1-like isoform X2 (XP_044471155.1), probable disease resistance protein At4g27220 (XP_044475559.1) and disease resistance protein At5g66890 isoform X1 (XP_044483347.1; **Supplementary Excel File 1**). Downregulated proteins in this pathway included cysteine-rich receptor-like protein kinase 10 (XP_044497351.1), PTI1-like tyrosine-protein kinase 1 isoform X1 (XP_044478686.1), and serine/threonine-protein kinase PBL27 isoform X1 (XP_044497851.1; **Supplementary Excel File 1**). Similarly, the upregulated proteins with the highest fold changes in the plant hormone signal transduction pathway included ethylene receptor (AAF61919.1), transcription factor BIM1-like isoform X1 (XP_044477274.1), and transcription factor LUX-like isoform X1 (XP_044505111.1; **Supplementary Excel File 1**). The downregulated proteins included two-component response regulator ARR2-like isoform X1 (XP_044484887.1) and scarecrow-like transcription factor PAT1 isoform X2 (XP_044501717.1; **Supplementary Excel File 1**). In the phenylpropanoid biosynthesis pathway, the upregulated proteins with the highest fold changes included 4-coumarate:CoA ligase 2 (AUZ94476.1), lignin-forming anionic peroxidase-like (XP_044483529.1), and peroxidase A2-like (XP_044483868.1), whereas the downregulated proteins included stemmadenine O-acetyltransferase-like (XP_044485770.1) and phenylacetaldehyde reductase (XP_044467159.1; **Supplementary Excel File 1**).

By 12 ad after Xcm infection, the proportion of downregulated DAPs had increased across nearly all annotated pathways in Aomang (**Supplementary Figure 8B**), resembling the directional pattern observed in Guifei leaves. The photosynthesis-antenna proteins pathway contained 14 downregulated DAPs and no upregulated DAPs. Carbon metabolism contained 104 downregulated DAPs and 31 upregulated DAPs, while the ribosome pathway contained 103 downregulated and 4 upregulated DAPs. The plant–pathogen interaction pathway also shifted toward a strong predominance of downregulated DAPs, with 88 downregulated and 8 upregulated proteins (**Supplementary Figure 8B**; **Supplementary Excel File 1**). These patterns indicated a transition from the early activation of several defense related pathways to a broader predominance of reduced protein abundance at 12 ad in Aomang leaves. Specifically, the downregulated DAPs assigned to the photosynthesis-antenna proteins pathway included chlorophyll a-b binding protein 151 (XP_044503929.1), chlorophyll a-b binding protein 3 (XP_044473400.1), photosystem I chlorophyll a/b-binding protein 3-1 (XP_044489557.1), and photosystem I chlorophyll a/b-binding protein 6 (XP_044464190.1; **Supplementary Excel File 1**). The upregulated proteins with the highest fold changes in the plant–pathogen interaction pathway at 12 ad of Xcm infection included putative serine/threonine-protein kinase-like protein CCR3 (XP_044482088.1), probable serine/threonine-protein kinase PBL28 (XP_044485169.1), LRR receptor-like serine/threonine-protein kinase IOS1 (XP_044485268.1), probable calcium-binding protein CML29 (XP_044479190.1), and calmodulin-7 (XP_044467398.1; **Supplementary Excel File 1**). The downregulated proteins included cysteine-rich receptor-like protein kinase 10 isoform X1 (XP_044482989.1), leucine-rich repeat receptor protein kinase HPCA1-like isoform X1 (XP_044461734.1), probable WRKY transcription factor 4 (XP_044468388.1), and calcium-dependent protein kinase 21-like (XP_044496846.1; **Supplementary Excel File 1**).

Overall, the cross-cultivar comparison revealed cultivar-specific differences in the early response to Xcm infection. Guifei showed a strong predominance of downregulated DAPs in core primary metabolic pathways from 6 ad onward, whereas Aomang showed early activation of several pathways, particularly the plant–pathogen interaction pathway at 6 ad before shifting toward extensive downregulation at 12 ad. In both cultivars, the 12 ad comparisons contained a greater predominance of downregulated DAPs than the corresponding 6 ad comparisons, indicating increasingly broad proteomic suppression during prolonged bacterial infection.

## 4 Discussion

Mango phyllosphere parts (e.g. leaf, stem, twig, flower, fruit) are prone to infection by diverse bacterial and fungal pathogens, causing substantial economic losses worldwide (Hussain et al., 2026). MBBS pathogen *X. citri* pv. *mangiferaeindicae* (Xcm) is predominantly prevalent in mango-growing regions worldwide, including China. The hemibiotrophic pathogen Xcm hijacks mango cell metabolism and represses immune responses through its secretion system and virulence effectors for host colonization and progressive disease formation. In the current study, the greater lesion diameter and disease index in Guifei cultivar confirm its higher susceptibility, whereas the lower symptom development in Aomang coincided with stronger late defense-enzyme activity. SOD converts O_2_^-^ to H_2_O_2_ but its induction was transient in Guifei and absent in Aomang; therefore, SOD alone did not explain the cultivar difference (Nunes da Silva et al., 2020; Rajput et al., 2021; Ramzan et al., 2021). In contrast, stronger POD activity and the late increase of CAT in Aomang suggest more effective H_2_O_2_ regulation, while class III peroxidases can also reinforce resistance through ROS control and cell wall lignifications (Li et al., 2020a). The stronger late PAL response in Aomang further supports phenylpropanoid-based production of phenolics and lignin, consistent with functional evidence linking PAL and 4CL activity to pathogen resistance (Solekha et al., 2020; Alariqi et al., 2023). We also observed a significant decline in the chlorophyll content of two mango cultivars (Guifei and Aomang) after 6 ad of Xcm infection. A decrease in chlorophyll content is directly associated with impaired photosynthetic activity and is considered a hallmark of pathogens (Lu and Yao, 2018). Pathogens reduce chlorophyll synthesis by disrupting chloroplast structure and function (Lu and Yao, 2018). We next observed a decrease in soluble sugars and reducing sugars after Xcm infection especially at the 12 ad sampling point, indicating an impaired carbon metabolism pathway. Sugars play a crucial role in plant immune responses because of their crucial role as energy sources and signaling molecules (Rojas et al., 2014). Furthermore, Xcm infection of mango leaves increased the levels of H_2_O_2_, O_2_^-^, and MDA at different time points, indicating lipid peroxidation and oxidative stress. Pathogen invasion and subsequent host recognition trigger an oxidative burst, stimulating plants to produce reactive oxygen species (ROS) as signaling molecules that facilitate the generation of antimicrobial agents (Castro-Moretti et al., 2020). However, excessive ROS accumulation in plant cells can cause oxidative damage to lipids, proteins, and nucleic acids (Sahu et al., 2022). Elevated MDA concentration detected in this study at 12d post-infection confirms peroxidative injury to cellular membranes, which in turn may disrupt cellular metabolic processes.

Our proteomic profiling revealed a number of differentially abundant proteins belonging to broad metabolic pathways, including carbon metabolism, photosynthesis, biosynthesis of amino acid, citrate cycle (TCA), phenylpropnoid biosynthesis, MAPK signaling pathway, and glutathione metabolism. The downregulation of the enriched carbon metabolism pathway in both cultivars after Xcm infection is a common response during plant-pathogen interaction due to the shift of resources from growth to defense (Rojas et al., 2014). The named DAPs support this interpretation in Guifei included reduced glyceraldehyde-3-phosphate dehydrogenases, cytosolic enolase 3, cytosolic pyruvate kinase-like and ornithine transcarbamylase indicate restricted carbon and amino acid metabolism. However, the Induction of glyoxylate/hydroxypyruvate reductase HPR3-like in carbon metabolism probably represents only partial compensation. We also observed downregulation of enriched photosynthesis-antenna proteins pathways in mango cultivars. The downregulation of chlorophyll a-b binding protein CP29.3, photosystem I chlorophyll a/b-binding protein 3-1, and chlorophyll a-b binding protein P4 was consistent with the observed chlorophyll decline. The photosynthesis pathway is important for the generation of ATP and carbohydrates, as it is involved in plant cell redox homeostasis during pathogen resistance (Liu et al., 2023; Zhang et al., 2025). Plants demand active photosynthesis during pathogen invasion to act as precursors for defense molecules, including salicylic and jasmonic acid, in order to activate the immune response (Hammerschmidt, 1999; Swarbrick et al., 2006). The significant downregulation of TCA in both Guifei and Aomang at 12d also indicate a systemic decrease in biosynthetic capacity and energy production. In addition, glutathione metabolism was also found to be significantly downregulated during pathogen infection in the Guifei cultivar. Glutathione is known to function in maintaining cell redox homeostasis and detoxifying ROS during pathogen stress (Gullner et al., 2018). Thus, the downregulation of glutathione metabolism likely exacerbates oxidative stress, impairing the mango’s ability to mitigate the excessive accumulation of ROS.

Interestingly, we observed DAPs in plant–pathogen interaction, plant hormone signal transduction, MAPK signaling pathway, and phenylpropanoid biosynthesis pathway in the Aomang cultivar at 6 ad of bacterial pathogen infection, which were then subsequently downregulated at 12 ad of pathogen infection. At the earlier 6 ad stage, Aomang showed greater accumulation of several defense-associated proteins, which together with lesion diameter and disease index suggest a comparatively stronger defense response than Guifei. The early Aomang response was supported by increased FERONIA, RPV1-like, disease resistance proteins At4g27220 and At5g66890, an ethylene receptor, BIM1-like and LUX-like transcription factor, consistent with receptor- and hormone-mediated immune activation (Stegmann et al., 2017; Yuan et al., 2021; Li et al., 2020b; Wang et al., 2021). Increased 4-coumarate:CoA ligase 2, lignin-forming anionic peroxidase-like, and peroxidase A2-like also connect the proteome with the stronger POD and PAL responses and with phenylpropanoid/cell-wall defense (Li et al., 2020a; Alariqi et al., 2023). However, reduced cysteine-rich receptor-like protein kinase, PTI1-like tyrosine-protein kinase, and serine/threonine-protein kinase PBL27 indicate selective rather than uniform activation of immune signaling. The MAPK signaling pathway is key to transducing pathogen-associated molecular patterns and activating host-defense-associated gene expression. Activation or suppression of the MAPK signaling pathway indicates that bacterial infection interferes with defense signal transduction pathways. Such mechanisms, where pathogen effectors target signaling pathways to dampen immunity have been reported for bacterial pathosystems (Timilsina et al., 2020). Next, secondary metabolism pathways, such as phenylpropanoid biosynthesis-associated DAPs, were also more abundant at 6d of bacterial infection, indicating the activation of the host plant resistance mechanism. Phenylpropanoids are sourced from phenylalanine, which includes benzenoids, coumarin, flavonoids, and lignin (Vogt, 2010). Previous studies have demonstrated that plant-derived phenolic compounds, especially flavonoids and coumarins, exert beneficial effects on the host by suppressing plant pathogens (Mandal et al., 2010; Ferreyra et al., 2012). Therefore, the activation and suppression of the phenylpropanoid biosynthetic pathway at 6d and 12d, respectively, indicates a biphasic mango-Xcm interaction, where early defensive phenylpropanoid production is countered by subsequent suppression of these pathways may be associated with prolonged pathogen. At 12 ad, serine/threonine-protein kinase CCR3, PBL28, IOS1, probable calcium-binding protein CML29 and calmodulin-7 remained induced. But leucine-rich repeat receptor protein kinase HPCA1-like, WRKY4, and calcium-dependent protein kinase 21-like decreased alongside broad pathway suppression. HCPA1 links H_2_O_2_ perception with calcium and systemic ROS signaling, and this pattern suggests weakening defense communication during prolonged infection (Fichman et al., 2022).

**Figure 8:**
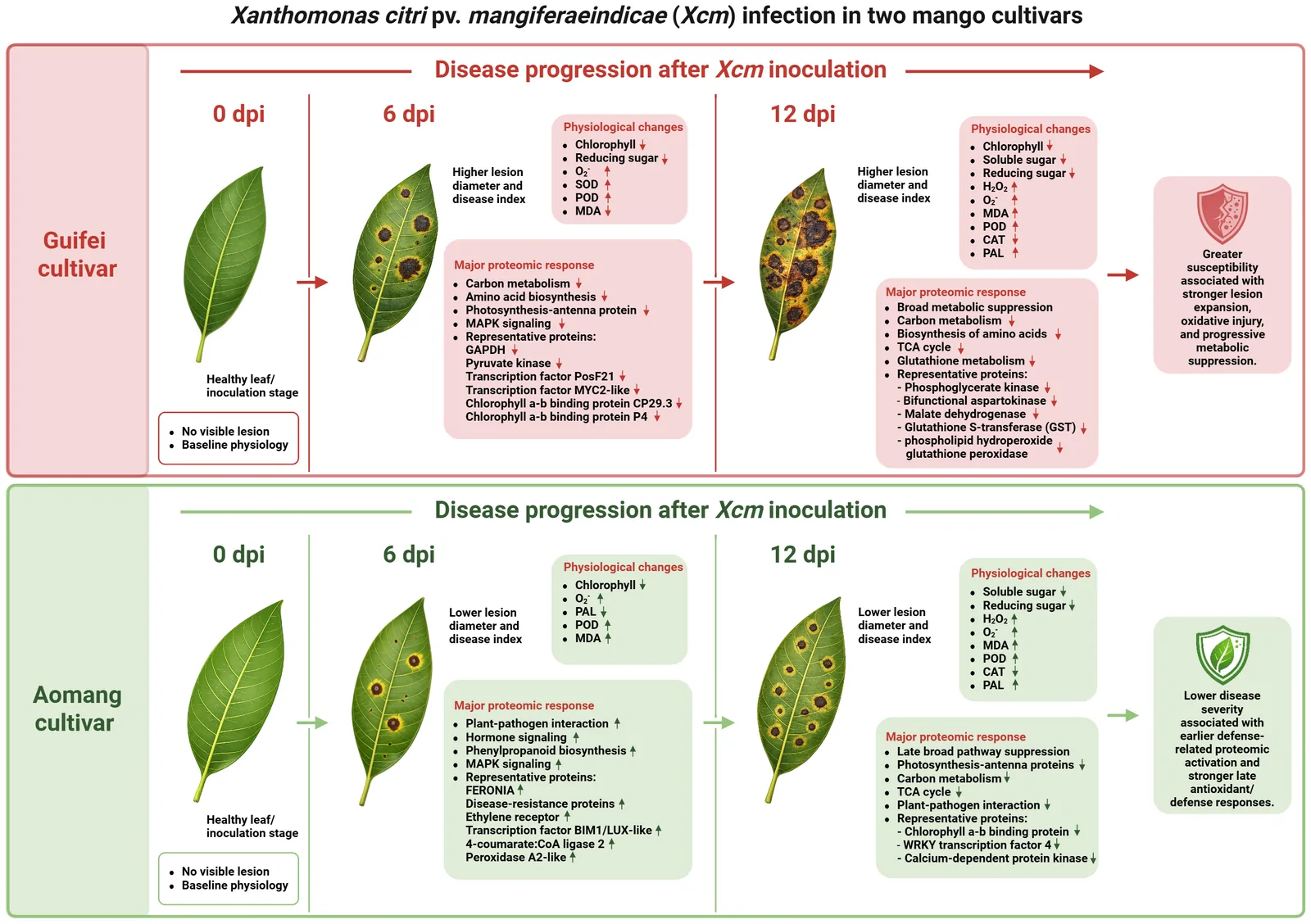
Schematic summary of cultivar-specific physiological and proteomic responses of mango cultivars during progression of bacterial black spot disease.

While both mango cultivars showed similar downregulation of key metabolic pathways, the extent and timing of pathway suppression varied significantly. In the Guifei cultivar, the core metabolism was downregulated more severely in the early phase, whereas the Aomang cultivar showed activation of some defensive pathways at 6 ad post-infection before widespread suppression occurred by 12 ad. The progressive decline in pathway activity from 6 to12 d reflects the cumulative impact of Xcm leaf colonization in response to increasing infection, which impaired metabolism and defense system, creating a cycle where the host ability to resist weakens over time (Cardoso et al., 2022). By combining physiological data and proteomic analysis, our study highlighted how these two mango cultivars responded to bacterial black spot disease across different time points, highlighting key shifts in metabolic pathways. Thus, earlier metabolic and redox disruption in Guifei versus earlier immune activation and stronger late POD, CAT and PAL activities in Aomang, provide a coherent explanation for their contrasting lesion severity (**Figure 8**). However, the late suppression in both cultivars nevertheless indicates incomplete resistance. In addition, the limitation of this study is that DAPs were not independently validated at the protein level. Therefore, the highlighted DAPs should be regarded as candidate components associated with cultivar-specific responses to Xcm, and their abundance and biological functions require further confirmation. Following functional validation, selected resistance- or susceptibility-associated candidates could be evaluated as molecular markers for MBBS-tolerant germplasm, incorporated into breeding programs, or explored as potential targets for gene-editing approaches such as CRISPR/Cas9.

## 5 Conclusion

This study demonstrates that Xcm caused progressive disease and extensive physiological and proteomic reprogramming in both mango cultivars but the magnitude and timing of these responses were cultivar dependent. Guifei developed larger lesions and a higher disease index, whereas Aomang showed lower disease severity together with stronger late POD, CAT, and PAL activities. Xcm infection reduced chlorophyll and sugar contents and promoted ROS accumulation and lipid peroxidation, confirming disruption of photosynthesis, carbon metabolism, and redox homeostasis. Proteomic profiling showed that Guifei underwent early suppression of glycolytic and photosynthetic proteins, followed by broader inhibition of TCA-cycle and glutathione-associated proteins. In contrast, Aomang exhibited early accumulation of immune- and defense-associated proteins, including FERONIA, disease-resistance proteins, hormone-signaling components and 4-coumarate:CoA ligase 2, before widespread pathway downregulation at 12 ad. Collectively, these findings indicate that Aomang possesses a comparatively stronger but incomplete defense-associated response, while Guifei is characterized by earlier metabolic and redox failure. The identified receptor, phenylpropanoid, antioxidant and redox-related proteins provide candidates for functional validation and resistance-oriented mango improvement.

## Data Availability

The datasets presented in this study can be found in online repositories. The names of the repository/repositories and accession number(s) can be found below: http://www.proteomexchange.org/, PXD079744.

## References

[B1] Alariqi M. Ramadan M. Wang Q. Yang Z. Hui X. Nie X. . (2023). Cotton 4‐coumarate‐CoA ligase 3 enhanced plant resistance to *Verticillium dahliae* by promoting jasmonic acid signaling‐mediated vascular lignification and metabolic flux. Plant J. 115, 190–204. 36994650 10.1111/tpj.16223

[B2] Bie F. Li Y. Liu Z. Qin M. Li S. Dan X. . (2022). High-quality genome resource of mango bacterial black spot pathogen Xanthomonas citri pv. mangiferaeindicae GXG07 isolated from Guangxi, China. Plant Dis. 106, 1027–1030. doi: 10.1094/pdis-08-21-1714-a 34633234

[B3] Cardoso J. L. Souza A. A. Vieira M. L. C. (2022). Molecular basis for host responses to *Xanthomonas* infection. Planta 256, 84. doi: 10.1007/s00425-022-03994-0 36114308

[B4] Castro-Moretti F. R. Gentzel I. N. Mackey D. Alonso A. P. (2020). Metabolomics as an emerging tool for the study of plant–pathogen interactions. Metabolites 10, 52. doi: 10.3390/metabo10020052 32013104 PMC7074241

[B5] Cheaib A. Killiny N. (2025). Photosynthesis responses to the infection with plant pathogens. Mol. Plant-Microbe Interact. 38, 9–29. doi: 10.1094/mpmi-05-24-0052-cr 39536275

[B6] Denova-Lozano P. Chamorro‐Flores A. Cerón‐Carpio A. B. Arroyo‐Becerra A. (2026). Determination of total soluble sugars in pteridophytes using the anthrone method. Curr. Protoc. 6, e70373. doi: 10.1002/cpz1.70373 42017451 PMC13101035

[B7] Ferreyra M. L. F. Rius S. P. Casati P. (2012). Flavonoids: biosynthesis, biological functions, and biotechnological applications. Front. Plant Sci. 3, 222. doi: 10.3389/fpls.2012.00222 23060891 PMC3460232

[B8] Fichman Y. Zandalinas S. I. Peck S. Luan S. Mittler R. (2022). HPCA1 is required for systemic reactive oxygen species and calcium cell-to-cell signaling and plant acclimation to stress. Plant Cell 34, 4453–4471. doi: 10.1093/plcell/koac241 35929088 PMC9724777

[B9] Gullner G. Komives T. Király L. Schröder P. (2018). Glutathione S-transferase enzymes in plant-pathogen interactions. Front. Plant Sci. 9, 1836. doi: 10.3389/fpls.2018.01836 30622544 PMC6308375

[B10] Guo Z. Yu Z. Li Q. Tang L. Guo T. Huang S. . (2021). *Fusarium* species associated with leaf spots of mango in China. Microb. Pathog. 150, 104736. 33453315 10.1016/j.micpath.2021.104736

[B11] Hammerschmidt R. (1999). Induced disease resistance: how do induced plants stop pathogens? Physiol. Mol. Plant Pathol. 55, 77–84. doi: 10.1006/pmpp.1999.0215 21632986

[B12] Hussain M. Adeel M. White J. C. (2025). Nano-selenium: a novel candidate for plant microbiome engineering. Trends Plant Sci. 30, 454–456. doi: 10.1016/j.tplants.2025.02.002 40011164

[B13] Hussain M. Ahmed N. Xie X. Teng Z. Hou X. Li X. . (2026). Characterization of the mango phyllosphere microbiome during fruit-setting unveils unique community assembly and coexisting beneficial-pathogenic microbiota. BMC Plant Biol. 26, 129. doi: 10.1186/s12870-025-07943-3 41419805 PMC12831448

[B14] Hussain M. Xuan P. Xin Y. Ma H. Zhou Y. Wen S. . (2024). Redundancy in microbiota-mediated suppression of the soybean cyst nematode. Microbiome 12, 125. doi: 10.1186/s40168-024-01840-x 39004755 PMC11247744

[B15] Kumar R. Kumar M. Chaudhary V. Teotia S. Singh D. (2025). Exploring recent advances, limitations, and future prospects of OMICS-based technologies in plant-pathogen interaction studies: a systematic review. Discov. Plant 2, 284. doi: 10.1007/s44372-025-00337-7 30311153

[B16] Li Q. Hu A. Qi J. Dou W. Qin X. Zou X. . (2020b). CsWAKL08, a pathogen-induced wall-associated receptor-like kinase in sweet orange, confers resistance to citrus bacterial canker via ROS control and JA signaling. Hortic. Res. 7, 42. doi: 10.1038/s41438-020-0263-y 32257228 PMC7109087

[B17] Li Q. Qin X. Qi J. Dou W. Dunand C. Chen S. . (2020a). CsPrx25, a class III peroxidase in Citrus sinensis, confers resistance to citrus bacterial canker through the maintenance of ROS homeostasis and cell wall lignification. Hortic. Res. 7, 192. doi: 10.1038/s41438-020-00415-9 33328465 PMC7705758

[B18] Liu F. Sun X. Wang L. Zhou K. Yao Q. Zhan R. L. (2023). Transcriptomic and proteomic analyses of Mangifera indica in response to *Xanthomonas critis* pv. *mangiferaeindicae*. Front. Microbiol. 14, 1220101. doi: 10.3389/fmicb.2023.1220101 37469435 PMC10352610

[B19] Lu Y. Yao J. (2018). Chloroplasts at the crossroad of photosynthesis, pathogen infection and plant defense. Int. J. Mol. Sci. 19, 3900. doi: 10.3390/ijms19123900 30563149 PMC6321325

[B20] Maldonado-Celis M. E. Yahia E. M. Bedoya R. Landázuri P. Loango N. Aguillón J. . (2019). Chemical composition of mango (*Mangifera indica* L.) fruit: Nutritional and phytochemical compounds. Front. Plant Sci. 10, 1073. doi: 10.3389/fpls.2019.01073 31681339 PMC6807195

[B21] Mandal S. M. Chakraborty D. Dey S. (2010). Phenolic acids act as signaling molecules in plant-microbe symbioses. Plant Signal. Behav. 5, 359–368. doi: 10.4161/psb.5.4.10871 20400851 PMC2958585

[B22] Nunes da Silva M. Vasconcelos M. W. Gaspar M. Balestra G. M. Mazzaglia A. Carvalho S. M. (2020). Early pathogen recognition and antioxidant system activation contributes to *Actinidia arguta* tolerance against *Pseudomonas syringae* pathovars *actinidiae* and *actinidifoliorum*. Front. Plant Sci. 11, 1022. doi: 10.3389/fpls.2020.01022 32793252 PMC7387506

[B23] Ouyang Q. Yang C. Lv L. Huang J. Li X. Zhu Z. (2025). The complete genome of *Xanthomonas citri* pv. *mangiferaeindicae* strain B3, isolated from diseased mango leaves in Guangxi, China. Microbiol. Resour. Announc. 14, e00299-25. doi: 10.1007/s12686-016-0657-1 40522699 PMC12243507

[B24] Patterson B. D. Macrae E. A. Ferguson I. B. (1984). Estimation of hydrogen peroxide in plant extracts using titanium (IV). Anal. Biochem. 139, 487–492. doi: 10.1016/0003-2697(84)90039-3 6476384

[B25] Rajput V. D. Harish Singh R. K. Verma K. K. Sharma L. Quiroz-Figueroa F. R. . (2021). Recent developments in enzymatic antioxidant defence mechanism in plants with special reference to abiotic stress. Biology 10, 267. doi: 10.3390/biology10040267 33810535 PMC8066271

[B26] Ramzan M. Sana S. Javaid N. Shah A. A. Ejaz S. Malik W. N. . (2021). Mitigation of bacterial spot disease induced biotic stress in Capsicum annuum L. cultivars via antioxidant enzymes and isoforms. Sci. Rep. 11, 9445. doi: 10.1038/s41598-021-88797-1 33941790 PMC8093210

[B27] Rojas C. M. Senthil-Kumar M. Tzin V. Mysore K. S. (2014). Regulation of primary plant metabolism during plant-pathogen interactions and its contribution to plant defense. Front. Plant Sci. 5, 17. doi: 10.3389/fpls.2014.00017 24575102 PMC3919437

[B28] Sahu P. K. Jayalakshmi K. Tilgam J. Gupta A. Nagaraju Y. Kumar A. . (2022). ROS generated from biotic stress: Effects on plants and alleviation by endophytic microbes. Front. Plant Sci. 13, 1042936. doi: 10.3389/fpls.2022.1042936 36352882 PMC9638130

[B29] Saqib A. A. N. Whitney P. J. (2011). Differential behaviour of the dinitrosalicylic acid (DNS) reagent towards mono-and di-saccharide sugars. Biomass Bioenergy 35, 4748–4750. doi: 10.1016/j.biombioe.2011.09.013 42574925

[B30] Shi J. Xie N. Zhang L. Pan X. Wang Y. Liu Z. . (2025). Optimizing row spacing and seeding rate for yield and quality of alfalfa in saline–alkali soils. Agronomy 15, 1828. doi: 10.3390/agronomy15081828 30654563

[B31] Solekha R. Susanto F. A. Joko T. Nuringtyas T. R. Purwestri Y. A. (2020). Phenylalanine ammonia lyase (PAL) contributes to the resistance of black rice against *Xanthomonas oryzae* pv. *oryzae*. J. Plant Pathol. 102, 359–365. doi: 10.1007/s42161-019-00426-z 30311153

[B32] Sossah F. L. Aidoo O. F. Dofuor A. K. Osabutey A. F. Obeng J. Abormeti F. K. . (2024). A critical review on bacterial black spot of mango caused by *Xanthomonas citri* pv. *mangiferaeindicae*: current status and direction for future research. For. Pathol. 54, e12860. doi: 10.1111/efp.12860 40046247

[B33] Stegmann M. Monaghan J. Smakowska-Luzan E. Rovenich H. Lehner A. Holton N. . (2017). The receptor kinase FER is a RALF-regulated scaffold controlling plant immune signaling. Science 355, 287–289. doi: 10.1126/science.aal2541 28104890

[B34] Swarbrick P. J. Schulze‐Lefert P. A. U. L. Scholes J. D. (2006). Metabolic consequences of susceptibility and resistance (race‐specific and broad‐spectrum) in barley leaves challenged with powdery mildew. Plant Cell Environ. 29, 1061–1076. doi: 10.1111/j.1365-3040.2005.01472.x 17080933

[B35] Testa R. Tudisca S. Schifani G. Di Trapani A. M. Migliore G. (2018). Tropical fruits as an opportunity for sustainable development in rural areas: The case of mango in small-sized Sicilian farms. Sustainability 10, 1436. doi: 10.3390/su10051436 30654563

[B36] Timilsina S. Potnis N. Newberry E. A. Liyanapathiranage P. Iruegas-Bocardo F. White F. F. . (2020). *Xanthomonas* diversity, virulence and plant–pathogen interactions. Nat. Rev. Microbiol. 18, 415–427. 32346148 10.1038/s41579-020-0361-8

[B37] Vogt T. (2010). Phenylpropanoid biosynthesis. Mol. Plant 3, 2–20. doi: 10.1093/mp/ssp106 20035037

[B38] Wang X. Li Y. Liu Y. Zhang D. Ni M. Jia B. . (2021). Transcriptomic and proteomic profiling reveal the key role of AcMYB16 in the response of *Pseudomonas syringae* pv. *actinidiae* in kiwifruit. Front. Plant Sci. 12, 756330. doi: 10.3389/fpls.2021.756330 34868148 PMC8632638

[B39] Xiaomei L. Wenbo L. Jinji P. Yixian X. Xin Z. Yanxiang Q. . (2009). Identification of resistance of mango cultivars against *Xanthomonas campestris* pv. *mangiferaeindicae*. J. Fruit Sci. 26, 349–352.

[B40] Xie X. Yang Z. Li D. Liu Z. Li X. Zhu Z. (2025). Proteomics analysis revealed the activation and suppression of different host defense components challenged with mango leaf spot pathogen *Alternaria alternata*. BMC Plant Biol. 25, 227. doi: 10.1186/s12870-025-06250-1 39972448 PMC11837451

[B41] Yuan M. Jiang Z. Bi G. Nomura K. Liu M. Wang Y. . (2021). Pattern-recognition receptors are required for NLR-mediated plant immunity. Nature 592, 105–109. doi: 10.1038/s41586-021-03316-6 33692546 PMC8016741

[B42] Zhang Q. Wang Z. Gao R. Jiang Y. (2025). Sugars, lipids and more: New insights into Plant Carbon sources during plant–microbe interactions. Plant Cell Environ. 48, 1656–1673. doi: 10.1111/pce.15242 39465686 PMC11695786

[B43] Zhou L. Liu X. Leng X. Zhang M. Yang Z. Xu W. . (2025). Genome-wide identification and expression analysis of the mango (*Mangifera indica* L.) SWEET gene family. Horticulturae 11, 675. doi: 10.3390/horticulturae11060675 30654563

