# Supplementary material for: Cultivar-specific physiological and proteomic patterns underlying mango susceptibility to bacterial black spot disease
Source: Front Plant Sci. 2026 Aug 25;17:1911657. doi: 10.3389/fpls.2026.1911657 (PMC13550206; doi:10.3389/fpls.2026.1911657)
Supplement: Supplementary file 1 [file DataSheet1.docx]

**Supplementary materials**

**Cultivar-Specific Physiological and Proteomic Patterns Underlying Mango Susceptibility to Bacterial Black Spot Disease**

Xiaona Xie^1,2,3^, Dongting Li^1,2,3^, Xianbin Hou^1,2,3^, Qiufei Ouyang^1,2^, Zhengjie Zhu^1,2^, Xi Li^1,2^, Zhengzhou Yang^1,2^, Muzammil Hussain^1,2,*^

^1^ Guangxi Key Laboratory of Biology for Mango, College of Agriculture and Food Engineering, Baise University, Baise 533000, China

^2^ University Engineering Research Center for Preservation and Comprehensive Utilization of Subtropical Characteristic Agricultural Products in Guangxi, College of Agriculture and Food Engineering, Baise University, Baise, China

^3^ Chongqing Three Gorges University, Chongqing, China

*Corresponding author’s email: muzammil0991@gmail.com (M. Hussain)

ORCID: 0000-0001-6681-7513 (M. Hussain)

Figure S1. Clustered heatmap of differentially abundant proteins from pathogen-infected and control Guifei leaves harvested at the 6-day sampling point.

Figure S2. Clustered heatmap of differentially abundant proteins from pathogen-infected and control Guifei leaves harvested at the 12-day sampling point.

Figure S3. Clustered heatmap of differentially abundant proteins from pathogen-infected and control Aomang leaves harvested at the 6-day sampling point.

Figure S4. Clustered heatmap of differentially abundant proteins from pathogen-infected and control Aomang leaves harvested at the 12-day sampling point.

Figure S5. Gene Ontology functional classification of differentially abundant proteins in pathogen-challenged versus control Guifei leaves at 6 d (A) and 12 d (B) post-inoculation. DAPs were grouped into Biological Process (BP), Cellular Component (CC), and Molecular Function (MF); orange = upregulated proteins, blue = downregulated proteins, with bar height indicating protein count per GO term.

Figure S6. Gene Ontology functional classification of differentially abundant proteins in pathogen-challenged versus control Aomang leaves at 6 d (A) and 12 d (B) post-inoculation. DAPs were grouped into Biological Process (BP), Cellular Component (CC), and Molecular Function (MF); orange = upregulated proteins, blue = downregulated proteins, with bar height indicating protein count per GO term.

Figure S7. Top enriched KEGG pathways of differentially abundant proteins (DAPs) from pathogen-challenged and control Guifei leaves at 6 d (A) and 12 d (B) post-inoculation. Red bars = upregulated protein number, blue bars = downregulated protein number; x-axis quantifies DAP counts per annotated pathway.

Figure S8. Top enriched KEGG pathways of differentially abundant proteins (DAPs) from pathogen-challenged and control Aomang leaves at 6 d (A) and 12 d (B) post-inoculation. Red bars = upregulated protein number, blue bars = downregulated protein number; x-axis quantifies DAP counts per annotated pathway.
